# Maternal arsenic exposure and gestational diabetes and glucose intolerance in the New Hampshire birth cohort study

**DOI:** 10.1186/s12940-016-0194-0

**Published:** 2016-11-08

**Authors:** Shohreh F. Farzan, Anala Gossai, Yu Chen, Lisa Chasan-Taber, Emily Baker, Margaret Karagas

**Affiliations:** 1 Division of Environmental Health, Department of Preventive Medicine, Keck School of Medicine of University of Southern California, 2001 N. Soto Street, MC 9237, Los Angeles, CA 90089 USA; 2 Department of Epidemiology, Geisel School of Medicine at Dartmouth, Lebanon, NH USA; 3 Department of Population Health, New York University School of Medicine, New York, NY USA; 4 Department of Biostatistics & Epidemiology, School of Public Health & Health Sciences, University of Massachusetts, Amherst, MA USA; 5 Department of Obstetrics and Gynecology, Geisel School of Medicine at Dartmouth, Lebanon, NH USA

**Keywords:** Arsenic, Gestational diabetes, Glucose intolerance, Pregnancy cohort, New Hampshire

## Abstract

**Background:**

Gestational diabetes mellitus (GDM) is a major pregnancy complication with detrimental effects for both mothers and their children. Accumulating evidence has suggested a potential role for arsenic (As) exposure in the development of GDM, but current studies have not assessed As exposure from water, urine or toenail samples.

**Methods:**

We investigated the association between As exposure and risk of glucose intolerance and GDM among 1151 women enrolled in the New Hampshire Birth Cohort Study. Arsenic was measured in home well water and via biomarkers (i.e., maternal urine collected ~24–28 weeks gestation and toenail clippings collected 2 weeks postpartum).

**Results:**

A total of 105 (9.1 %) of women were diagnosed with glucose intolerance and 14 (1.2 %) of women were diagnosed with GDM. A total of 10.3 % of women had water As levels above 10 μg/L, with a mean As level of 4.2. Each 5 μg/L increase in As concentration in home well water was associated with a ~10 % increased odds of GDM (OR: 1.1, 95 % CI 1.0, 1.2). A positive and statistically significant association also was observed between toenail As and GDM (OR: 4.5, 95 % CI 1.2, 16.6), but not urinary arsenic (OR: 0.8, 95 % CI 0.3, 2.4). In a stratified analysis, the association between water As and GDM and glucose intolerance was largely limited to obese women (OR: 1.7, 95 % CI 1.0, 2.8).

**Conclusions:**

Our findings support the role of As exposure via water from private wells in the incidence of GDM and that this association may be modified by body composition.

**Electronic supplementary material:**

The online version of this article (doi:10.1186/s12940-016-0194-0) contains supplementary material, which is available to authorized users.

## Background

Gestational diabetes mellitus (GDM), the onset or first recognition of diabetes during pregnancy, is a pregnancy complication on the rise, with reported increases of nearly 50 % in the US from 1990 to 2009 [1]. The Centers for Disease Control estimated that glucose intolerance affects approximately 9.2 % of pregnancies in the United States annually [2]. Women who develop GDM are at higher risk for birth and delivery complications, including infant macrosomia, neonatal hypoglycemia, and cesarean delivery [3, 4]. Further, later life risks for both mothers and their children have been documented. Women who develop GDM have nearly a 7-fold increased risk of type 2 diabetes in the decade after delivery and children born to mothers with GDM are also more likely to develop glucose intolerance and cardiometabolic disease later in life [5–9].

While the rise in GDM is likely due to a number of causes, exposure to environmental contaminants, such as toxic metals, may be a possible contributor. Arsenic (As) is a widespread, naturally occurring contaminant, to which millions worldwide are exposed primarily via contaminated water sources [10]. In our study area of New Hampshire, USA, approximately 40 % of households rely on unregulated private water systems, of which 10–15 % contain As levels exceeding the US EPA maximum contaminant level of 10 μg/L [11]. While As is most commonly known for its carcinogenic effects, it also has been associated with a number of deleterious health effects including cardiovascular disease, respiratory disease, immune impairment and glucose intolerance in adults [10]. A number of studies have explored the link between As exposure and diabetes (reviewed in [12–14]) and experimental studies have proposed several mechanisms whereby As could affect β-cell function and disrupt glucose homeostasis, including endocrine disruption, glucose uptake and transport, gluconeogenesis, adipocyte differentiation, epigenetic effects and oxidative stress [14, 15]. Further, studies of vulnerable populations, including pregnant women and children, suggest they may be particularly susceptible to As’s harmful effects [16–20].

Although few epidemiological studies have examined As exposure and GDM, data are beginning to emerge on the potential role for As in the development of impaired gestational glucose control [21–23]. Specifically, only three prior studies have examined the association between As and GDM, and of these, two were limited in that they relied solely on postpartum As exposure assessments. Here in our current study, we assessed the relationship between GDM and As levels in three different matrices (water, urine and toenails), which together provide a robust combination of both short- and long-term exposure measures. For example, toenail As is a useful biomarker for determining prolonged exposure to inorganic As, as its affinity for keratin’s sulfhydryl groups causes As to accumulate in nails. Given the slow growth rate of toenails, toenail clippings can reflect an exposure window approximately 6 to 12 months prior to collection [24]. Therefore, toenail samples that collected from recently postpartum mothers can be thought to represent As exposure early in pregnancy. Lastly, none of the prior studies evaluated the potential of effect modification by pre-pregnancy body mass index (BMI). A number of recent studies have indicated that pre-pregnancy obesity may put women at a higher risk of developing complications during their pregnancy, including GDM [25–28]. Therefore, we evaluated the association between water, urinary, and toenail As and risk of GDM and glucose intolerance among women enrolled in the New Hampshire Birth Cohort Study, and whether this association was modified by pre-pregnancy BMI.

## Methods

### The New Hampshire birth cohort

The New Hampshire Birth Cohort is an ongoing study and starting in January 2009, we began recruiting 18–45 year old pregnant women receiving prenatal care at study clinics, as previously described [29]. Women were enrolled at 24–28 weeks gestation if they reported using water from a private well at their residence since their last menstrual period and were not planning to move prior to delivery. Only singleton births are included in the study.

### Medical record review and glucose testing

Participants completed a detailed medical history and lifestyle questionnaire upon enrollment and a follow-up questionnaire at 2 weeks postpartum to obtain updated information about changes in key exposures and prenatal complications. After delivery, participants’ medical records were reviewed to abstract health information, including glucose challenge test (GCT) results, oral glucose tolerance test (OGTT) results, diagnoses of GDM, hypertension, pre-eclampsia and eclampsia. Other clinical information was recorded to verify self-reported medical and reproductive history.

The GCT is typically administered to pregnant women between 24 and 28 weeks gestation to screen for GDM. The patient is given a standard solution containing 50 g of glucose to drink and after 1 h, a blood sample is obtained to test for blood glucose. If GCT results indicate possible glucose intolerance (i.e. a blood glucose level of 120 to 140 mg/dL is considered borderline, 140 to <200 mg/dL is considered a positive result and above 200 mg/dL is considered a high positive result (based upon the recommendations of the American College of Obstetricians and Gynecologists), then an OGTT is typically performed to confirm a GDM diagnosis [30, 31]. In cases where the GCT indicates a high positive result (>200 mg/dL), the physician typically will make the diagnosis of GDM without performing the additional OGTT. The OGTT is a diagnostic test performed to confirm GCT results. The patient provides a baseline pre-test blood sample, and then drinks a standard solution containing 100 g of glucose. Blood samples are collected at timed intervals of 1, 2, and 3 h, at which blood glucose is monitored and given a positive or negative score based on the expected rate of clearance at each timepoint, based on the diagnostic guidelines of the American Diabetes Association [30, 32].

Based on results of GCT and OGTT testing, we categorized women as normal, glucose intolerant or GDM. Specifically, women were classified with glucose intolerance if they had either: 1) a borderline (120 to <140 mg/dL) or positive (140 to <200 mg/dL) GCT result and a follow-up OGTT with at least one positive score, or 2) a positive GCT result (140 to <200 mg/dL) but no follow-up OGTT. Women were classified with GDM if they had either 1) a positive GCT result (140 to <200 mg/dL) and a follow-up OGTT with 2 or more positive scores or 2) a positive GCT result (140 to <200 mg/dL) and a physician diagnosis of GDM in the medical record or 3) a high positive GCT result (>200 mg/dL). Women were categorized as “normal” if they had either 1) a negative GCT result or 2) a borderline or positive GCT result (120 to <200 mg/dL) and subsequent follow-up OGTT with negative scores throughout the test. This set of classification criteria was necessary for two reasons: 1) in cases where a GCT result is 200 mg/dl or higher, the physician may make the diagnosis of GDM without performing a subsequent OGTT and 2) as there are no clinically defined criteria for glucose intolerance, we used the information from the GCT and OGTT to investigate those women who may have failed their initial GCT screening and had one or more above normal glucose readings during their OGTT, but may not have met the criteria for a diagnosis of GDM. We did not reclassify any women who had been physician diagnosed by OGTT with GDM as normal or glucose intolerant. This method resulted in mutually exclusive categories of GDM, glucose intolerant or normal.

We also constructed a continuous GCT variable, which reflected the 24–28 week GCT result in mg/dL, if available. Some patients underwent early and multiple testing, thus the average of all GCT (mg/dL) results within the 24–28 week gestational age range was reported. If the patient was not tested within the 24–28 week gestational age range, the GCT result from the test closest to 24–28 weeks was reported. Tests that were conducted before 22.5 weeks or after 32.5 weeks were excluded.

### Arsenic exposure assessment

Participants were given instructions and prepaid mailing materials upon enrollment to collect samples of their home tap water and return the samples to the study office, which were analyzed by inductively coupled plasma mass spectrometry (ICP-MS) at the Dartmouth Trace Element Analysis Core, as previously described [29]. Women provided a spot urine sample upon enrollment at 24–28 weeks gestation, which was collected and stored, as previously described [29]. Urine samples were analyzed for levels of arsenite (iAs^III^), arsenate (iAs^V^), monomethylarsonic acid (MMA), dimethylarsinic acid (DMA) and arsenobetaine by high-performance liquid chromatography (HPLC) inductively coupled plasma mass spectrometry (ICP-MS) at the University of Arizona Hazard Identification Core [33–35]. Total urinary As was calculated by summing inorganic (iAs = iAs^III^ + iAs^V^) and organic (DMA, MMA) metabolites [29]. Arsenobetaine, a form of As found in seafood was excluded, as it is thought to pass through the body unmetabolized [36]. Maternal toenail samples were collected at 2 weeks postpartum. Women were asked to remove any nail polish, clip a normal amount of toenail from each toe after bathing and place clippings into a small collection envelope. Samples were washed 5 times by sonication in a solution of Triton X-100 and acetone, followed by deionized water and then dried before low-pressure microwave digestion. Samples were analyzed for trace elements using ICP-MS as previously described for As [37]. Toenail samples collected from women in the study weighed ~25 mg on average, while a minimum of 1–2 mg of nail is typically needed for analysis. Arsenic was detected in 90 % of maternal toenails and 84.4 % of water samples. Water As detection limits ranged 0.001–0.07 μg/L. Urine samples that registered below the As detection limit (ranging from 0.10 to 0.15 μg/L for individual urine species; 0.5, 16.8, and 36.6 % of the study population were below the detection limit for DMA, MMA and iAs, respectively) were assigned a value equal to the detection limit divided by the square root of two. Approximately 56 % of women had detectable levels of all metabolites. As secondary exposure measures, we constructed primary (PMI) and secondary methylation indices (SMI) from ratios of MMA to iAs and DMA to MMA in urine, respectively, as these are considered indicators of methylation capacity that may impact individual variability in health effects of As exposure [38].

### Statistical analysis

We confined our analysis to women without a history of diabetes and who had one or more exposure measurements for As in well water, toenail clippings, and/or urine. Arsenic exposure variables were modeled as continuous variables and were untransformed, with the exception of toenail As, for which natural log-transformation was necessary for model convergence. First, we used multinomial logistic regression models, adjusted for potential confounders, to test the association between As exposure variables (water As, ln-toenail As, urinary As) and a 3-level outcome variable of GDM, glucose intolerance, and normal blood glucose, to distinguish between levels of disease severity. Next, we used logistic regression models, adjusted for potential confounders, to test the relation between As exposure variables (each modeled separately) and either glucose intolerance only or GDM only in order to investigate the independent associations for each outcome. Then, we used logistic regression models, adjusted for potential confounders, to test the relation between As exposure variables (each modeled separately) and with a combined outcome variable, where both glucose intolerance and GDM cases were pooled, in order to examine all cases of any glucose control impairment together. Lastly, we also used linear regression models, adjusted for potential confounders, to test the relation between As exposure variables and continuous GCT values.

We examined the As exposures of interest with GDM outcomes in both unadjusted and minimally adjusted models (enrollment age and educational attainment) (Additional file 1: Table S1). Our final models were adjusted for covariates available from medical records and questionnaires that could potentially influence glucose control based on a priori considerations, including age at enrollment, pre-pregnancy body mass index (BMI), pregnancy weight gain, smoking during pregnancy, secondhand smoke exposure during pregnancy, educational attainment, and gestational week of glucose testing. In models where urinary arsenic was the exposure of interest, we additionally adjusted for urinary creatinine in our analyses.

Potential effect modification by pre-pregnancy BMI was evaluated by inspection of stratum specific odds ratios and by including a multiplicative interaction term in the multivariable logistic regression models and assessing its statistical significance at *p* < 0.05 using likelihood ratio chi-square tests. In stratified analyses, maternal pre-pregnancy BMI was categorized by World Health Organization standards [39].

## Results

As of May 1, 2016, a total of 1473 participants had enrolled in the NHBCS and of those, 1307 had GCT and/or OGTT results available in their medical record. An additional 30 women were excluded due to a history of diabetes. Of those, 1151 women had at least one available As exposure measurement for either urine, toenails and/or water at the time of analysis, resulting in final sample sizes of 853 (water As), 778 (toenail As) and 1049 (urinary As).

A total of 14 (1.2 %) women were diagnosed with GDM and 105 (9.1 %) women were categorized as glucose intolerant. The mean (SD) GCT level was 110.7 (26.3) mg/dL.

Nearly 1 in every 10 households (88 of 853 available samples; 10.3 %) tested in this study had water As levels above EPA maximum contaminant limit of 10 μg/L, with a mean As level of 4.2 μg/L (range: 0.001–189.3 μg/L). Women in this sample had a mean toenail As concentration of 0.1 μg/g, with values ranging from 0.001 to 0.7 μg/g and a mean urinary As concentration of 5.9 μg/L with values ranging from 0.2 to 288.5 μg/L (Table 1). Water As (above 1 μg/L) was positively correlated with both toenail As (*r* = 0.61, *p* < 0.0001) and urinary As (*r* = 0.35, *p* < 0.0001), but water As below 1 μg/L was not (toenail As *r* = 0.08, *p* = 0.11 and urinary As *r* = −0.01, *p* = 0.81). Urinary As also correlated with toenail As (*r* = 0.18, *p* < 0.0001). Statistically significant differences between women with normal glucose control and those with either glucose tolerance or GDM were observed for enrollment age, race, pre-pregnancy BMI and weight gain during pregnancy (Table 1).

**Table 1:** Characteristics for 1151 pregnant women enrolled in the New Hampshire Birth Cohort Study with at least one exposure measurement

	*N* (%) or mean (SD)		
Variable	Normal (*n* = 1032)	Glucose Intolerant & GDM (*n* = 119)	*p*-value**
Maternal age at enrollment	30.9 (4.9)	32.2	0.02
Race			
White	1020 (98.8)	113 (95.0)	0.01
Educational attainment			
Less than college	304 (32.9)	32 (32.4)	
College graduate	362 (39.2)	43 (43.4)	
Any post-graduate school	258 (27.9)	24 (24.2)	0.48
Relationship status			
Married	787 (85.2)	90 (90.9)	
Single, separated, divorced	137 (14.8)	9 (9.1)	0.28
Pre-pregnancy BMI*	25.7 (5.6)	28.2 (6.3)	<0.0001
Weight gain during pregnancy*	35.3 (14.5)	28.9 (16.3)	<0.0001
Parity*			
Nulliparous	428 (41.5)	48 (40.3)	
1 to 2	530 (51.4)	61 (51.3)	
3+	74 (7.2)	10 (8.4)	0.53
Smoke Exposure			
Ever smoked during pregnancy	61 (6.5)	4 (4.0)	0.67
Exposed to secondhand smoke during pregnancy	131 (13.9)	14 (14.0)	0.77
Arsenic Exposure			
Well water arsenic (μg/L)	4.2 (13.2); range 0.001–189.3	4.1 (13.0); range 0.005–96.5	0.83
Well water ≥10 μg/L MCL	81 (10.7)	7 (7.5)	0.48
Urinary arsenic (μg/L)	5.8 (12.4); range 0.2–288.5	6.5 (15.5); range 0.6–157.3	0.10
Toenail arsenic (μg/g)	0.1 (0.1); range 0.001–0.7	0.1 (0.1); range 0.009–0.7	0.50
Glucose Control			
Glucose Intolerant	0	105	
Gestational diabetes	0	14	

*Missing (*n*)- pre-pregnancy BMI (41), weight gain (42), smoking (110), secondhand smoke (108), school level (128), relationship status (128), urinary arsenic (102), well water arsenic (298), toenail arsenic (373). **Compared using the *X* ^2^ test or Fisher’s exact test (categorical variables) and Wilcoxon rank sum test (for continuous variables)

Using multinomial regression models adjusted for enrollment age, education, smoking during pregnancy, secondhand smoke exposure during pregnancy, pre-pregnancy BMI, weight gain during pregnancy, and gestational week of glucose testing, we observed a borderline statistically significant association between water As and GDM, with a 10 % increased risk of GDM associated with each 5 μg/L increase in water As (OR: 1.1, 95 % CI: 1.0, 1.2) (Table 2). We also observed a statistically significant positive association between toenail As and GDM, with each 100 % increase in toenail As associated with nearly a four-fold increased risk of GDM (OR: 4.5, 95 % CI: 1.2, 16.6) (Table 2), but with wide confidence intervals. We did not observe statistically significant associations urinary As and GDM (OR: 0.8, 95 % CI: 0.3, 2.4) nor between any of the arsenic measures and risk of glucose intolerance (Table 2). We also examined the association of As exposure with the each of the outcomes individually (glucose intolerance only or GDM only; Additional file 1: Table S2). These results mirrored those observed with the multinomial models, such that for each 5 μg/L increase in water As we observed a marginally significant increase risk of GDM only (OR: 1.1, 95 % CI: 1.0, 1.2). We also found that each 100 % increase in toenail As was associated with a statistically significant increased risk of GDM only (OR: 4.5, 95 % CI: 1.3, 16.2), but with wide confidence intervals (Additional file 1: Table S2). We did not observe any relationship between of any of the As exposure variables and glucose intolerance only, nor did we observe an association of any measure of As exposure with the pooled outcome of any glucose intolerance (i.e. combined glucose intolerance and GDM) (Table 2). Furthermore, we did not observe any relationship between of any of the As exposure variables and GCT, when GCT results were used as a continuous measure (Additional file 1: Table S3).

**Table 2:** Relation between arsenic exposure and glucose tolerance status

		Glucose Intolerant^a^		Gestational Diabetes Mellitus^a^		Combined Glucose Intolerant and GDM^b^	
Arsenic exposure	Non-cases	Cases	OR (95 % CI)	Cases	OR (95 % CI)	Cases	OR (95 % CI)
Water As^†^	663	79	1.0 (0.9, 1.1)	11	1.1 (1.0, 1.2)*	90	1.0 (0.9, 1.1)
Urinary As^†^	582	61	1.0 (1.0, 1.1)	9	0.8 (0.3, 2.4)	70	1.0 (1.0, 1.1)
Toenail As^‡^	616	62	0.9 (0.6, 1.3)	7	4.5 (1.2, 16.6)**	69	0.9 (0.7, 1.3)

^a^Outcome variable is a 3-level variable (0 = normal, 1 = intolerant, 2 = GDM), modeled using multinomial regression; ^b^combined glucose intolerance and GDM is modeled with logistic regression using a dichotomous variable (0 = normal or 1 = glucose intolerant and GDM). Models were adjusted for enrollment age, education, smoking during pregnancy, secondhand smoke exposure during pregnancy, pre-pregnancy BMI, weight gain and gestational week of glucose testing. Additional adjustment for urinary creatinine was included in the urinary As model only. ^†^Per 5 μg/L increase in water or urinary As; ^‡^per 100 % increase in toenail As; *0.1 < *p* < 0.05; **p <0.05

Next, we evaluated whether pre-pregnancy BMI modified the association between As and glucose intolerance (Table 3). Among women who were categorized as obese (BMI ≥ 30 kg/m^2^), for each 5 μg/L increase in water As we observed a statistically significant increase risk of GDM or glucose intolerance (OR: 1.7, 95 % CI: 1.0, 2.8; *p*-value = 0.04) (Table 3). In contrast, there were no associations between As and GDM/glucose intolerance among women who were underweight to normal or overweight. We tested for interaction between water As and maternal BMI, using a multiplicative interaction term in our model, but this did not reach statistical significance (p-interaction = 0.08).

**Table 3:** Relation between water arsenic concentrations^†^ and combined endpoint of GDM or glucose intolerance (pooled), stratified by pre-pregnancy BMI

Pre-pregnancy BMI	Non-cases	Cases	% Cases^‡^	OR (95 % CI)
Underweight to Normal (<25)	388	32	8.2	0.9 (0.8, 1.2)
Overweight (25–29.99)	157	25	15.9	1.0 (0.8, 1.2)
Obese (≥30)	118	33	28.0	1.7 (1.0, 2.8)**

Combined outcome variable was coded a dichotomous variable (0 = normal or 1 = GDM or intolerant). Models were adjusted for enrollment age, education, smoking during pregnancy, secondhand smoke exposure during pregnancy, weight gain and gestational week of glucose testing. ^†^Per 5 μg/L increase in water As; ^‡^Represents the percent of total cases within each BMI category; **p <0.05

Lastly, we performed additional sensitivity analyses to test the relationship between arsenic exposure and GDM, among those with water As above and below the 10 μg/L MCL. When we restricted our analyses to the only those individuals with water As below 10 μg/L, we found that the association between water As and GDM remained positive (OR: 1.4, 95 % CI: 0.2–9.4), albeit with wide confidence intervals. Similarly, when we examined the relation between toenail As and GDM among those with water As below 10 μg/L, we see a similar positive trend (GDM OR: 2.6, 95 % CI: 0.6–11.4; Intolerant OR: 0.9, 95 % CI: 0.6–1.3), but also with wide confidence intervals. We were unable to test only those with water As above 10 μg/L, due to limited sample size and few cases of GDM or glucose intolerance with water As above 10 μg/L.

## Discussion

In this prospective study of US women, we evaluated the associations between multiple markers of As exposure and glucose intolerance and GDM during pregnancy. We observed a positive statistically significant association between toenail As and GDM. We also found that each 5ug/L increase in As in a woman’s household well water was related to nearly a 10 % greater risk of GDM, which was consistent with our findings for toenail As. There was no evidence that higher levels of maternal urinary As was related to greater risks of either glucose intolerance during pregnancy or GDM. We only observed an association for As exposure and GDM, but did not find evidence of an association with glucose intolerance. Although one may assume that these are two conditions on the same spectrum of metabolic dysfunction, it is possible that there may be differences in underlying pathophysiology, which could be differentially impacted by As exposure. Further analyses stratified by pre-pregnancy BMI indicate that these associations may be primarily limited to obese women, who made up approximately 37 % of total combined cases of glucose intolerance or GDM.

Our findings are consistent with those of prior studies that have observed a relationship between As and glucose control during pregnancy. In one of the first studies to examine the role of As in glucose tolerance, Ettinger et al. conducted a cross-sectional study of 532 women living near the Tar Creek Superfund site in Oklahoma. The authors found that the highest quartile of maternal blood As at delivery was associated with impaired glucose tolerance at 24–28 weeks of gestation (OR: 2.8, 95 % CI: 1.1, 6.9) [21]. They also reported that hair As, a long-term biomarker that is similar to toenail As, was associated with a 4-fold increased odds of impaired glucose tolerance (OR: 4.2, 95 % CI: 0.7–23.9), although this was not statistically significant [21]. These results were similar to our observation of an association between toenail As and GDM. In a large longitudinal study of 1274 Canadian women, Shapiro et al. observed a positive relationship between the highest quartile of first trimester blood As and GDM (OR: 3.7, 95 % CI: 1.4, 9.6), as well as with combined GDM and glucose intolerance (OR: 1.9, 95 % CI: 1.1, 3.5) [22]. A nested case–control study within pregnancy cohort of Chinese women compared metals, including As, in meconium samples from infants born to healthy mothers (*n* = 190) or mothers with GDM (*n* = 137). Meconium As was dose-dependently associated with maternal GDM (OR: 3.3, 95 % CI: 1.2, 8.7; OR: 3.4, 95 % CI: 1.3, 8.8; and OR: 5.3, 95 % CI: 2.0, 13.9, for the 2nd, 3rd and 4th quartiles of meconium As, respectively) [23].

The various As biomarkers have a number of strengths and limitations, which impact how the data are interpreted. For example, water As levels are a good surrogate for As exposure where the primary source of exposure is water and where individual water intake levels are known, but will underestimate exposure levels among populations with high inorganic As intake from foods, such as rice [29, 40]. Arsenic measured in blood tends to have a very short half-life, which may make it more prone to exposure misclassification than more stable, long-term measures [41]. Blood As measurements are also typically not speciated for As metabolites and total blood As includes inorganic and organic species that may vary in their toxicity. Thus, blood As measures may overestimate exposure in some individuals who consume fish and seafood, primary sources for organoarsenicals, which are thought to pass through the body unmetabolized and may or may not contribute to overall toxicity [36]. However, in populations that are chronically exposed to As, blood As can reach a steady-state and reflect long-term exposure levels [42]. Urinary As is a short-term biomarker of both inorganic and organic As species and generally reflects exposure from both water and food sources over the past 2–3 days [42], although studies have found that urinary As concentrations over a period of time remain within a relatively constant range in adults [43, 44]. Toenail As, like hair As used in previous studies, is a biomarker of inorganic As exposure that approximates a window of exposure ~6–12 months prior to sample collection, providing a more long-term exposure measure than urine [24]. For example, in the case of our current study, toenail samples collected from mothers at 2 weeks postpartum can be thought to represent a period of exposure in early pregnancy. We only observed associations for toenail As with GDM and marginal associations with water As. Given that water As comprised only of inorganic As and toenails primarily reflect inorganic As levels, it is possible that differences in the associations between the three As biomarkers in our study with GDM may suggest that inorganic As, in contrast to organic As, may be more important in the development of GDM. However, we cannot distinguish between intake of organic As and metabolism of inorganic As to organic As metabolites in urine samples and when we examined urinary levels of inorganic versus organic As species, neither were related to GDM or glucose intolerance alone (data not shown). It is possible that there is additional residual confounding that we may not have accounted for in this context and we may not have been able to detect an effect with urinary As due to the fact that only 56 % of individuals had detectable levels of all urinary As species. Furthermore, urinary As levels may not be a reliable indicator of exposure when examining a metabolic disease that may impact urinary dilution.

Several studies have suggested that women with greater pre-pregnancy BMI may be at higher risk of developing GDM, but to our knowledge, our study is the first to explore maternal pre-pregnancy BMI as a potential effect modifier of the relationship between As and GDM [26–28]. In an analysis of combined GDM and glucose intolerance, our results suggested that As exposure and glucose intolerance outcomes may be more strongly related among obese women, although this was of borderline statistical significance. Further analyses of GDM or glucose intolerance as individual outcomes were limited by our sample size.

The question of whether environmental contaminants may impact GDM risk is one of critical importance. In addition to possible associations with As, exposure to air pollutants, cadmium and chromium have also been associated with GDM and glucose intolerance [45–47]. GDM is the most common medical complication of pregnancy and places women at a higher risk of caesarean section, birth injuries due to infant macrosomia, and neonatal mortality [48]. Maternal hyperglycemia increases fetal insulin levels and stimulates growth, leading to higher birth weights among children born to mothers with GDM [48, 49]. Among the long-term consequences of GDM, evidence suggests that mothers who experience GDM are more likely to develop Type II diabetes mellitus later in life [5]. Children born to mothers with GDM exhibit impaired fetal endothelial function and growing evidence indicates that these children have greater risks of developing cardiometabolic disease, obesity and Type II diabetes [6–9, 50, 51]. While further investigation of As’s role in development of GDM is needed, increases in the risk of GDM could impact long-term health of both mothers and their children.

A major strength of our study is the use of multiple As exposure markers that reflect different types of exposure at various points within pregnancy, as well as the ability to adjust for detailed medical history and sociodemographic information. Our study had limited statistical power to observe some associations with GDM. Although nearly 10 % met criteria for glucose intolerance, only 1 % of women were diagnosed with GDM, in contrast to US national GDM prevalence estimates, which range from ~3 to 10 % of all pregnancies [2], although these estimates may vary based upon prenatal glucose screening thresholds [31]. However, in spite of this limitation we observed a statistically significant association between toenail As and GDM and a marginal association for home well water As and risk of GDM. Further larger investigations are needed and may consider contribution of other risk factors such as diet, physical activity and variant polymorphisms in genes related to glucose control, which may increase the risk of type II diabetes [52], as well as poor glucose regulation during pregnancy.

## Conclusions

Our findings support a potential role for As exposure via water sources in the development of GDM, but not glucose intolerance. Given the relatively high prevalence of exposure to As through water and food sources, even small exposure-related increases in the risk of GDM could have long-term health ramifications for both mothers and their children. Further characterization of modifiable environmental risk factors could significantly improve the long-term health of both pregnant women and their children.

## Additional file

Additional file 1: Supplementary Tables. (DOCX 18 kb)

## Abbreviations

As: Arsenic
BMI: Body mass index
GCT: Glucose challenge test
GDM: Gestational diabetes mellitus
OGGT: Oral glucose tolerance test
