# Supplementary material for: Maternal arsenic exposure and gestational diabetes and glucose intolerance in the New Hampshire birth cohort study
Source: Environ Health. 2016 Nov 8;15:106. doi: 10.1186/s12940-016-0194-0 (PMC5101688; doi:10.1186/s12940-016-0194-0)
Supplement: Additional file 1: — Supplementary Tables. (DOCX 18 kb) [file 12940_2016_194_MOESM1_ESM.docx]

| **Table S1. Relation between arsenic exposure and glucose tolerance status.** | | | | | |
| --- | --- | --- | --- | --- | --- |
|  |  | **Glucose Intolerant** | | **Gestational Diabetes Mellitus** | |
| **Arsenic exposure** | **Non-cases** | **Cases** | **OR (95% CI)** | **Cases** | **OR (95% CI)** |
| **Model 1** | | | | | |
| Water As^†^ | 760 | 82 | 1.0 (0.9, 1.1) | 11 | 1.1 (1.0, 1.2) |
| Urinary As^†^ | 947 | 91 | 1.0 (1.0, 1.1) | 11 | 0.9 (0.5, 1.6) |
| Toenail As^‡^ | 707 | 64 | 0.9 (0.6, 1.2) | 7 | 1.5 (0.6, 3.8) |
| **Model 2** | | | | | |
| Water As^†^ |  |  | N/A |  | N/A |
| Urinary As^†^ | 670 | 64 | 1.0 (1.0, 1.1) | 9 | 0.8 (0.3, 2.0) |
| Toenail As^‡^ |  |  | N/A |  | N/A |
| **Model 3** | | | | | |
| Water As^†^ | 760 | 82 | 1.0 (0.9, 1.1) | 11 | 1.1 (1.0, 1.2) |
| Urinary As^†^ | 670 | 64 | 1.0 (1.0, 1.1) | 9 | 0.7 (0.3, 2.1) |
| Toenail As^‡^ | 707 | 64 | 0.9 (0.7, 1.2) | 7 | 1.6 (0.6, 4.2) |
| **Model 4** | | | | | |
| Water As^†^ | 663 | 79 | 1.0 (0.9, 1.1) | 11 | 1.1 (1.0, 1.2)* |
| Urinary As^†^ | 831 | 88 | 1.0 (1.0, 1.1) | 9 | 0.8 (0.3, 2.4) |
| Toenail As^‡^ | 616 | 62 | 0.9 (0.6, 1.3) | 7 | 4.5 (1.2, 16.6)** |
| Outcome variable is a 3-level variable (0=normal, 1=intolerant, 2=GDM), modeled using multinomial regression. Model 1 is unadjusted; model 2 is adjusted for urinary creatinine only (urinary As model only); Model 3 was adjusted for age and educational attainment (and urinary creatinine in the urinary As model only); Model 4 was adjusted for Model 3 variables, as well as smoking during pregnancy, secondhand smoke exposure during pregnancy, pre-pregnancy BMI, weight gain and gestational week of glucose testing.  ^†^Per 5 μg/L increase in water or urinary As; ^‡^per 100% increase in toenail As; *0.1<p<0.05; **p <0.05. | | | | | |

| **Table S2. Relation between arsenic exposure and GDM or glucose intolerance, modeled as independent outcomes.** | | | |
| --- | --- | --- | --- |
|  | **Non-cases** | **Cases** | **OR (95% CI)** |
| **GDM Only** | | | |
| Water As^†^ | 663 | 11 | 1.1 (1.0, 1.2)* |
| Urinary As^†^ | 582 | 9 | 0.9 (0.3, 2.4) |
| Toenail As^‡^ | 616 | 7 | 4.5 (1.3, 16.2)** |
| **Glucose Intolerant Only** | | | |
| Water As^†^ | 663 | 79 | 1.0 (0.9, 1.1) |
| Urinary As^†^ | 582 | 61 | 1.0 (1.0, 1.1) |
| Toenail As^‡^ | 616 | 62 | 0.8 (0.6, 1.2) |
| Outcome variable was coded a dichotomous variable (0=normal or 1=GDM for GDM only models; 0=normal or 1=intolerant for glucose intolerant only models). Models were adjusted for enrollment age, education, smoking during pregnancy, secondhand smoke exposure during pregnancy, pre-pregnancy BMI, weight gain and gestational week of glucose testing. Additional adjustment for urinary creatinine was included in the urinary As model only. ^†^Per 5 μg/L increase in water or urinary As; ^‡^per 100% increase in toenail As; *0.1<p<0.05; **p <0.05. | | | |

| **Table S3. Relation between arsenic exposure and average glucose challenge testing result (mg/dL).** | | |
| --- | --- | --- |
|  | **N** | **β (95% CI)** |
| Water As^†^ | 771 | -0.4 (-1.1, 0.3) |
| Urinary As^†^ | 675 | 0.1 (-0.6, 0.7) |
| Toenail As^‡^ | 720 | -0.9 (-3.4, 1.6) |
| Outcome variable of average glucose testing result is a continuous measurement, modeled using linear regression models adjusted for age, educational attainment smoking during pregnancy, secondhand smoke exposure during pregnancy, pre-pregnancy BMI, weight gain and gestational week of glucose testing. Additional adjustment for urinary creatinine was included in the urinary As model only. ^†^Per 5 μg/L increase in water or urinary As; ^‡^per 100% increase in toenail As. | | |
